# A new genus and ten new species of spiders (Arachnida, Araneae) from Iran

**DOI:** 10.3897/zookeys.1054.70408

**Published:** 2021-08-03

**Authors:** Alireza Zamani, Yuri M. Marusik

**Affiliations:** 1 Zoological Museum, Biodiversity Unit, University of Turku, FI-20014, Turku, Finland University of Turku Turku Finland; 2 Institute for Biological Problems of the North RAS, Portovaya Str.18, Magadan 685000, Russia Institute for Biological Problems of the North RAS Magadan Russia; 3 Department of Zoology & Entomology, University of the Free State, Bloemfontein 9300, South Africa University of the Free State Bloemfontein South Africa

**Keywords:** Middle East, new combination, *
Sestakovaia
*, taxonomy

## Abstract

One new genus (*Sestakovaia***gen. nov.**; Liocranidae) and 10 new species of five families of spiders are described from different provinces of Iran: *Brigitteaavicenna***sp. nov.** (♂♀, Alborz and Kurdistan provinces) (Dictynidae), *Micariaatropatene***sp. nov.** (♂, East Azerbaijan Province), *Zagrotesborna***sp. nov.** (♂, Hormozgan Province), *Z.parla***sp. nov.** (♂, Kerman Province) (Gnaphosidae), *Sestakovaiahyrcania***sp. nov.** (♂, Golestan Province), *Mesioteluspatricki***sp. nov.** (♂, Golestan Province) (Liocranidae), *Palpimanuscarmania***sp. nov.** (♂, Kerman Province), *P.persicus***sp. nov.** (♂♀, Hormozgan Province) (Palpimanidae), *Rhysodromusgenoensis***sp. nov.** (♂, Hormozgan Province), and *R.medes***sp. nov.** (♂, Hormozgan Province) (Philodromidae). Furthermore, *Sestakovaiaannulipes* (Kulczyński, 1897), **comb. nov.** (ex. *Mesiotelus*) and *Zagrotesbifurcatus* (Zamani, Chatzaki, Esyunin & Marusik, 2021), **comb. nov.** (ex. *Berinda*) are proposed as new combinations.

## Introduction

Currently, 890 species in 321 genera and 54 families of spiders are known from Iran (Zamani et al. 2021b; unpublished data). Although there have been many recent taxonomic revisions and large-scale faunistic surveys focusing on Iranian spiders (e.g., Montemor et al. 2020; Zamani and Bosselaers 2020; Zamani et al. 2020; Zamani and Marusik 2021a, b; Zamani et al. 2021a), the araneofauna of Iran nevertheless remains inadequately known, with much of the country poorly sampled and new species and distribution records found regularly. In this paper we contribute to the knowledge about spiders in Iran by describing a new genus and 10 new species, raising the number of spider species known from this country to 900 species in 322 genera.

## Material and methods

Specimens were photographed using a Canon EOS 7D camera attached to an Olympus SZX16 stereomicroscope and a JEOL JSM-5200 scanning electron microscope at the Zoological Museum of the University of Turku. Specimens were photographed in dishes with paraffin on the bottom holding the specimens in desired position. Digital images were montaged using CombineZP and Helicon focus 3.10 image stacking software programs and edited using CorelDraw graphic design software. Lengths of palp and leg segments were measured on the dorsal side and are listed as: total length (femur, patella, tibia, metatarsus [missing on the palp], tarsus).

### Abbreviations

**ALE** anterior lateral eye;

**AME** anterior median eye;

**PLE** posterior lateral eye;

**PME** posterior median eye;

**RTA** retrolateral tibial apophysis.

### Depositories (with curators’ names in parentheses)

**MHNG**Muséum d’histoire naturelle, Genève, Switzerland (Peter J. Schwendinger);

**NHMW**Naturhistorisches Museum Wien, Vienna, Austria (Christoph Hörweg).

## Taxonomy

### Family Dictynidae O. Pickard-Cambridge, 1871

#### Genus Brigittea

Taxon classificationAnimaliaAraneaeDictynidae

Lehtinen, 1967

0150A706-A198-52EB-B414-9ADFE041A757

##### Comments.

*Brigittea* is a small genus with six nominal species distributed in the Western Palaearctic, all of which were previously classified in *Dictyna* Sundevall, 1833. Males have a highly elevated cephalic region and chelicerae with lateral condyles and deeply concaved mesal margins. Females differ from those of the related genera by their spaced receptacles (vs touching each other).

#### Brigittea avicenna sp. nov.

Taxon classificationAnimaliaAraneaeDictynidae

92FF2DB6-EC5E-5485-ADC4-0C6A4CCDB13A

http://zoobank.org/D03A116C-9C98-48B0-BD23-B8B36B6EC07E

**Figure 1.**
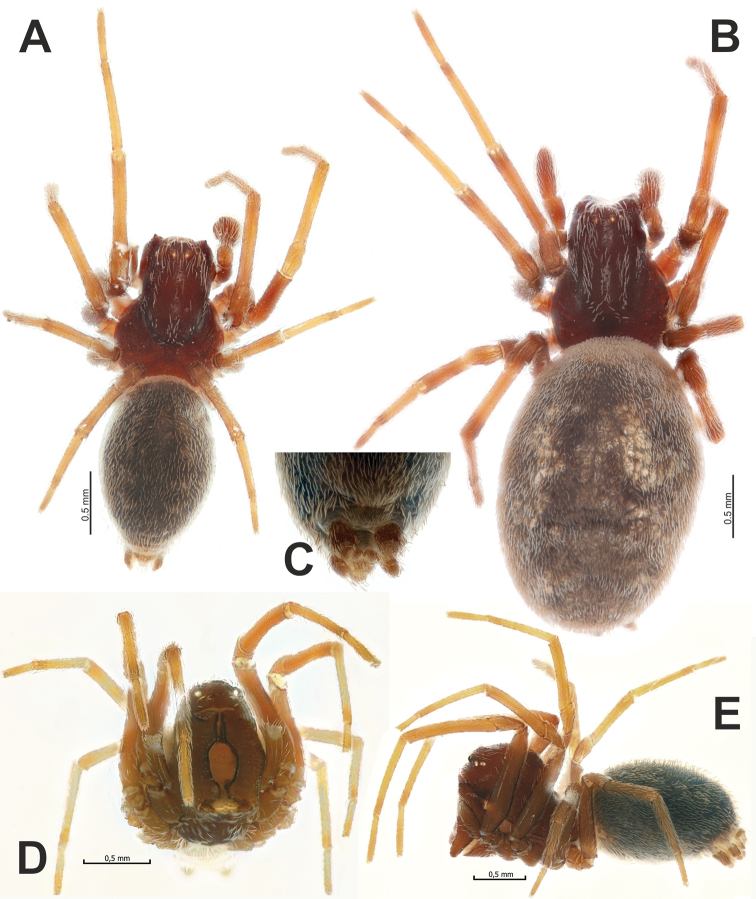
Male (**A, C–E**) and female (**B**) of *Brigitteaavicenna* sp. nov. **A, B** habitus, dorsal **D, E** same, frontal and lateral, respectively **C** cribellum and spinnerets, ventral.

**Figure 2.**
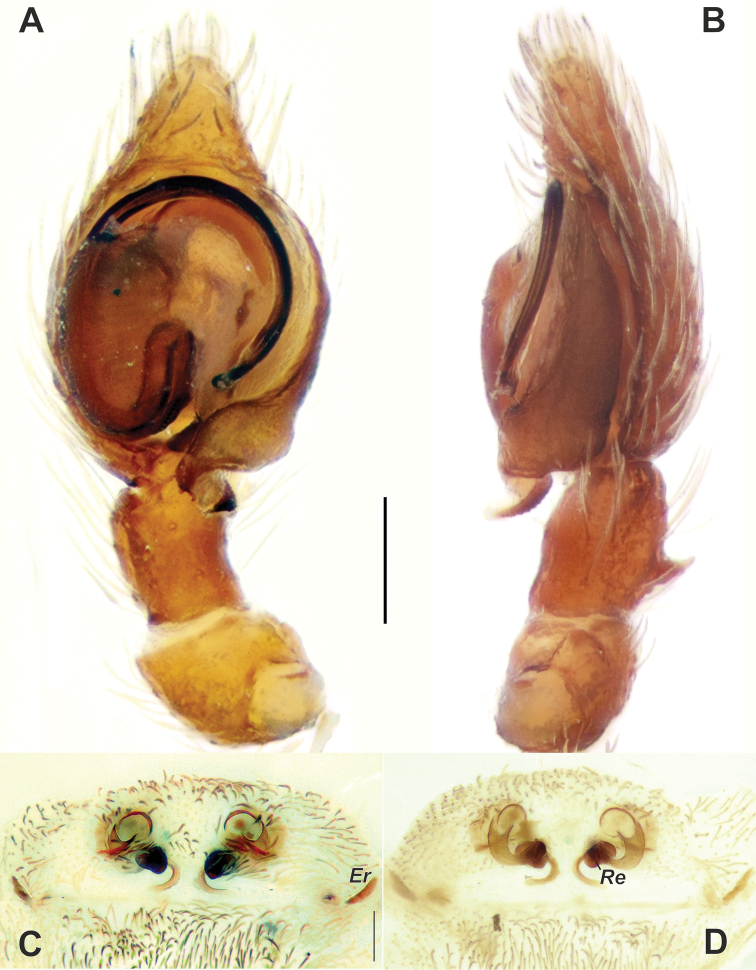
Male palp (**A, B**) and epigyne (**C, D**) of *Brigitteaavicenna* sp. nov. **A, C** ventral **B** retrolateral **D** dorsal. Abbreviations: *Er* – epigynal ridge, *Re* – receptacle. Scale bars: 0.2 mm.

**Figure 3.**
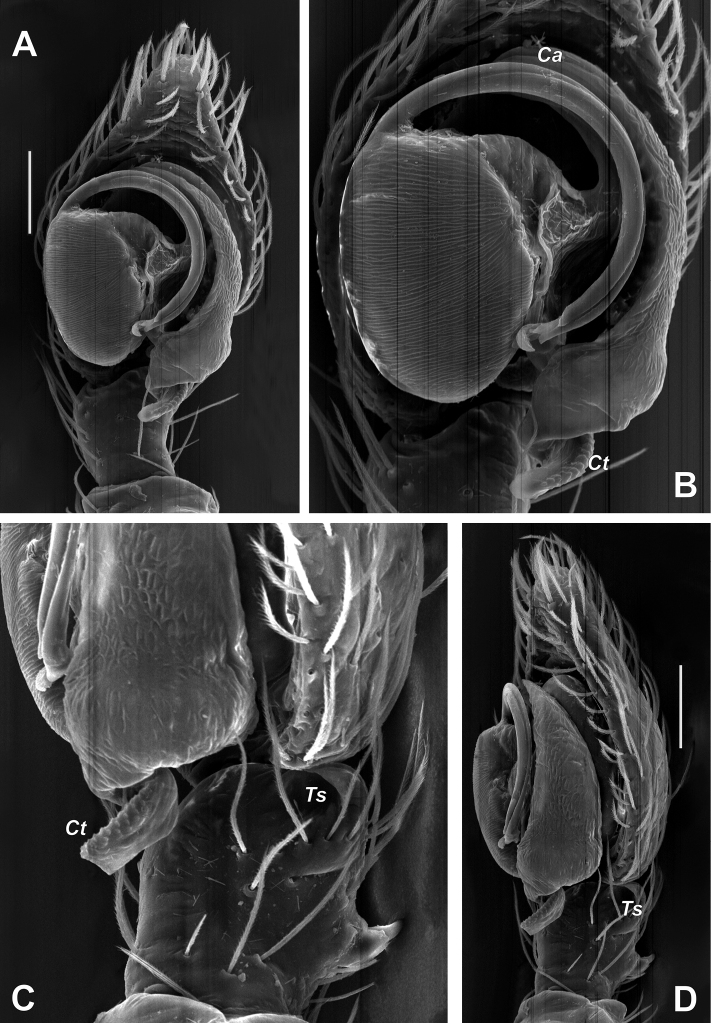
SEM images of the male palp of *Brigitteaavicenna* sp. nov. **A, B** ventral **C, D** retrolateral. Abbreviations: *Ca* – anterior part of conductor, *Ct* – posterior part of conductor, *Ts* – tibial socket. Scale bars: 0.1 mm.

Figures 1A–E , 2A–D , 3A–D

##### Type material.

***Holotype*** ♂ (MHNG), Iran: Kurdistan Province: S of Divandareh, 35°45'N, 47°05'E, 23.6.1975 (A. Senglet). ***Paratypes***: 3♀ (MHNG), same collection data as the holotype; 2♀ (MHNG), Alborz Province: Asara, 36°02'N, 51°14'E, 1900 m, 4.7.1975 (A. Senglet).

##### Etymology.

The new species is named after Ibn Sina, also known as Avicenna (ca 980–June 1037), a Persian polymath who is regarded as the father of early modern medicine; noun in apposition.

##### Diagnosis.

The male of the new species differs from all congeners by its thick embolus (vs filamentous) and the posterior tip of conductor (*Ct*) directed mesally (vs retrolaterally). The female can be distinguished by the almost round copulatory openings and ridges (*Er*) spaced by more than 8 diameters of copulatory openings (vs copulatory openings not round and ridges spaced by less than 3 diameters).

##### Description.

**Male.** Habitus as in Figure 1A, D, E. Total length 2.60. Carapace 1.25 long, 0.55 wide at pars cephalica, 0.85 at pars thoracica, pars cephalica very high, higher than ½ (ca 0.65) of carapace length. Eye sizes: AME: 0.05, ALE: 0.06, PME: 0.06, PLE: 0.06. Carapace, labium, chelicera, maxilla and sternum dark reddish brown, without any pattern. Pars cephalica and sternum with sparse coating of long white setae. Legs light brown, without annulations. Abdomen dark grey, with coating of dense short white setae. Spinnerets brownish, unicolourous. Cribellum undivided (Fig. 1C). Measurements of legs: I: 3.50 (1.04, 0.34, 0.85, 0.81, 0.46), II: 3.07 (0.94, 0.33, 0.70, 0.68, 0.42), III: 2.23 (0.71, 0.27, 0.42, 0.50, 0.33), IV: 2.55 (0.80, 0.29, 0.54, 0.59, 0.33).

Palp as in Figures 2A, B, 3A–D; tibia with dorso-retrolateral socket (*Ts*); cymbium almost 2 times longer than wide; anterior part of conductor (*Ca*) terminates at about 11:30 o’clock position; posterior part (*Ct*) covered with fine granulation, tip directed mesally; embolic base large, about ½ of cymbium’s length; embolus thick, originates at 10 o’clock position, tip modified with fine hook terminally.

**Female (*paratype* from Kurdistan).** Habitus as in Figure 1B. Total length 3.60. Carapace 1.15 long, 0.58 wide at pars cephalica, 0.94 at pars thoracica. Coloration, general somatic features and eye sizes as in male. Measurements of legs: I: 3.57 (1.06, 0.40, 0.77, 0.79, 0.55), II: 3.09 (0.96, 0.36, 0.63, 0.71, 0.43), III: 2.36 (0.75, 0.33, 0.46, 0.52, 0.30), IV: 3.02 (0.95, 0.39, 0.66, 0.68, 0.34).

Epigyne as in Figure 2C, D; epigynal field about 3 times wider than long; copulatory opening almost round, spaced by 1.6 diameters, lateral ridges (*Er*) located 2 diameters of copulatory openings apart from copulatory openings, their length about diameter of copulatory openings; copulatory ducts gradually tapering, making a course of about 90°, about 2 times longer than copulatory opening’s diameter; receptacles (*Re*) smaller than copulatory openings, spaced slightly by more than one width.

##### Comments.

At first look, the male palp is similar to those of the species of *Emblyna* Chamberlin, 1948 due to the modified embolus (thick and with complex tip). Current generic placement is due to the strongly raised cephalic region, which is also documented in the generotype, *Brigittealatens* (Fabricius, 1775), and modification of chelicera (cf. Fig. 1D and Miller and Svatoň 1978: pl. IV, figs 1, 2). All species assigned to *Brigittea* similarly have the posterior tip of conductor covered with fine teeth (cf. Marusik et al. 2015: figs 31, 35). Another similarity with the generotype is spaced receptacles (vs touching each other in *Emblyna* and *Dictyna*). Although *B.latens* has a filamentous embolus, its tip is modified in a similar way as in *B.avicenna* sp. nov. (cf. Marusik et al. 2015: fig. 36). There is another character that is different in the new species and the generotype – the structure of the cribellum. In the new species the cribellum is integral, while in *B.latens* it is bipartite (cf. Lehtinen 1967).

##### Distribution.

Known only from the listed localities in Alborz and Kurdistan provinces, northern and western Iran.

### Family Gnaphosidae Pocock, 1898

#### Genus Zagrotes

Taxon classificationAnimaliaAraneaeGnaphosidae

Zamani, Chatzaki, Esyunin & Marusik, 2021

795F8D5A-70C9-5ACC-8E72-FDD94D4DE30A

##### Comments.

This genus was recently described as monotypic from southwestern and southern Iran, with *Zagrotesapophysalis* Zamani, Chatzaki, Esyunin & Marusik, 2021 as the type species. In the same paper, Zamani et al. (2021a) described another similar species in another genus, namely *Berindabifurcata* Zamani, Chatzaki, Esyunin & Marusik, 2021. The two new species described here are closely related to *B.bifurcata*, and to a lesser degree to *Z.apophysalis*. All species possess a bifurcated RTA and similar conformation of the bulb. For this reason, we propose a new combination, *Zagrotesbifurcatus* (Zamani, Chatzaki, Esyunin & Marusik, 2021), comb. nov., despite the fact that this species and the two new ones described here lack modifications on palpal patella and swollen tibia that are present in the type species.

##### Composition.

Four species: *Z.apophysalis*, *Z.borna* sp. nov., *Z.bifurcatus* comb. nov., and *Z.parla* sp. nov.

##### Distribution.

Endemic to Iran, distributed along the southwestern and southern slopes of Zagros Mountains, from Kohgiluyeh and Boyer-Ahmad to Hormozgan.

#### Zagrotes borna sp. nov.

Taxon classificationAnimaliaAraneaeGnaphosidae

A7F8C52D-9103-52B1-B2CE-74E97BE9497F

http://zoobank.org/DA557472-6803-4C50-A165-BF60F41828AC

**Figure 4.**
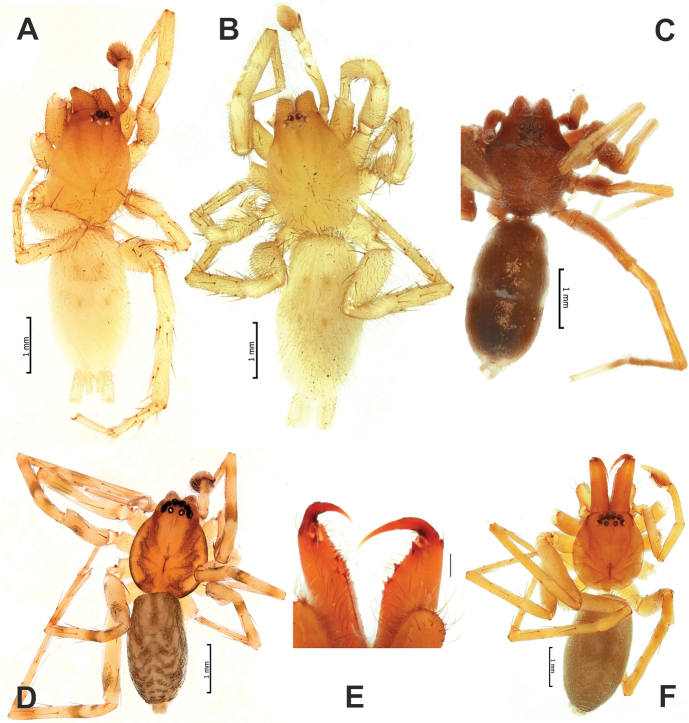
Males of *Zagrotesborna* sp. nov. (**A**), *Z.parla* sp. nov. (**B**), *Micariaatropatene* sp. nov. (**C**), *Sestakovaiahyrcania* sp. nov. (**D**) and *Mesioteluspatricki* sp. nov. (**E, F**) **A–D, F** habitus, dorsal **E** chelicerae, ventral. Scale bars: 0.2 mm, unless stated otherwise.

**Figure 5.**
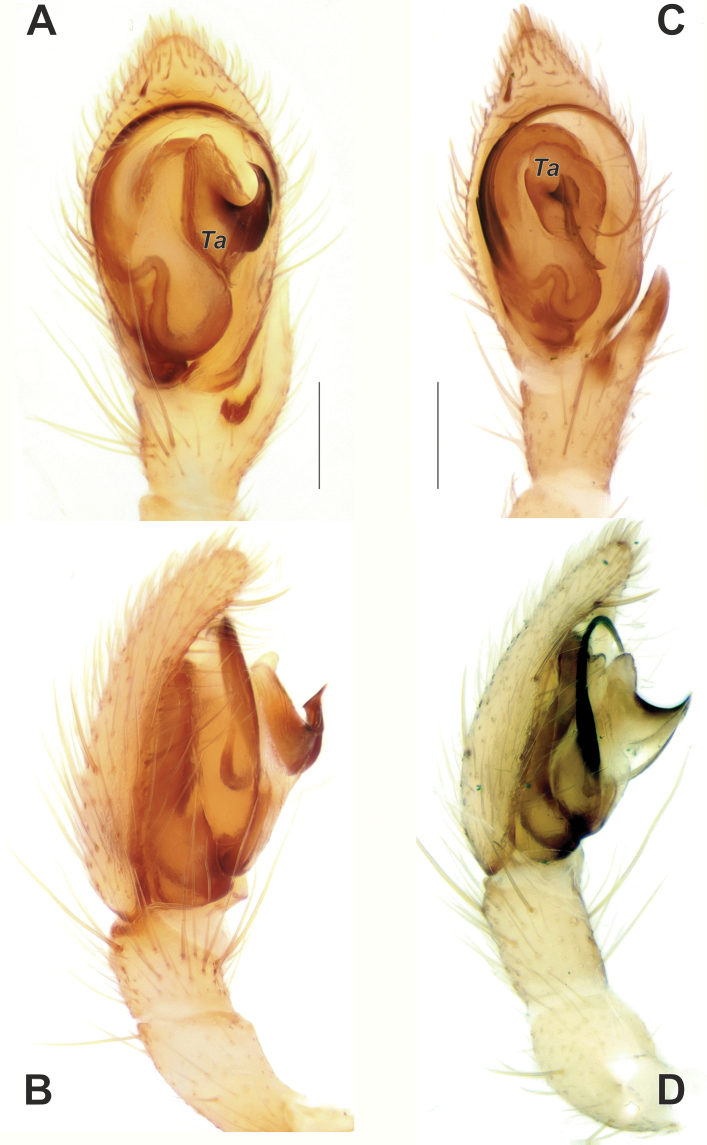
Male palps of *Zagrotesborna* sp. nov. (**A, B**) and *Z.parla* sp. nov. (**C, D**) **A, C** ventral **B, D** prolateral. Abbreviation: *Ta* – tegular apophysis. Scale bars: 0.2 mm.

**Figure 6.**
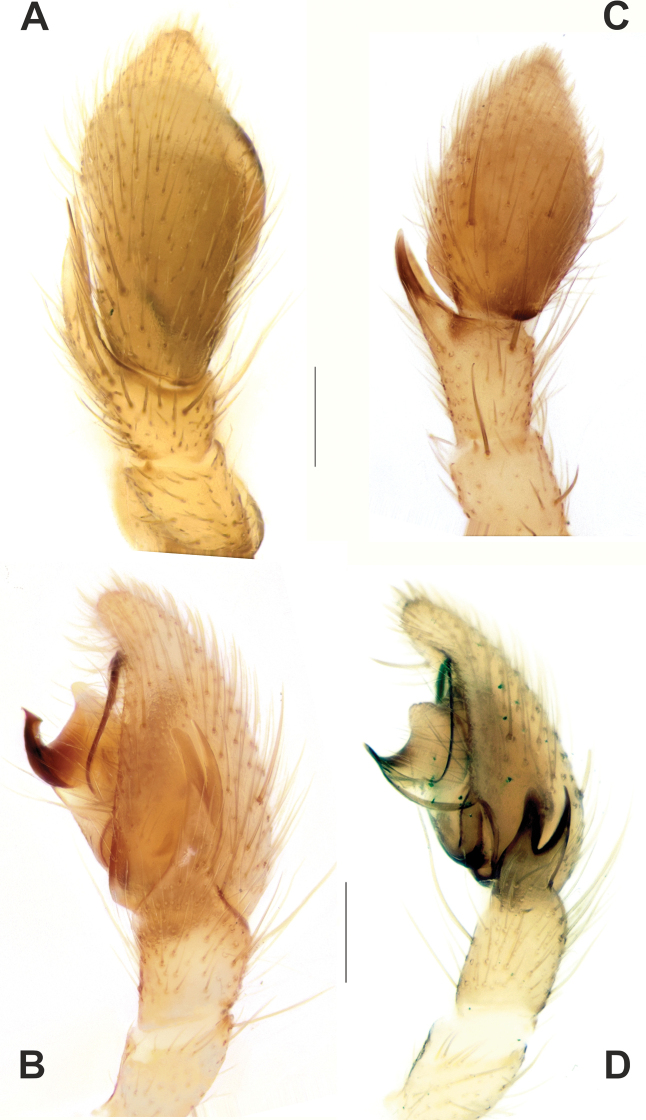
Male palps of *Zagrotesborna* sp. nov. (**A, B**) and *Z.parla* sp. nov. (**C, D**) **A, C** dorsal **B, D** retrolateral. Scale bars: 0.2 mm.

**Figure 7.**
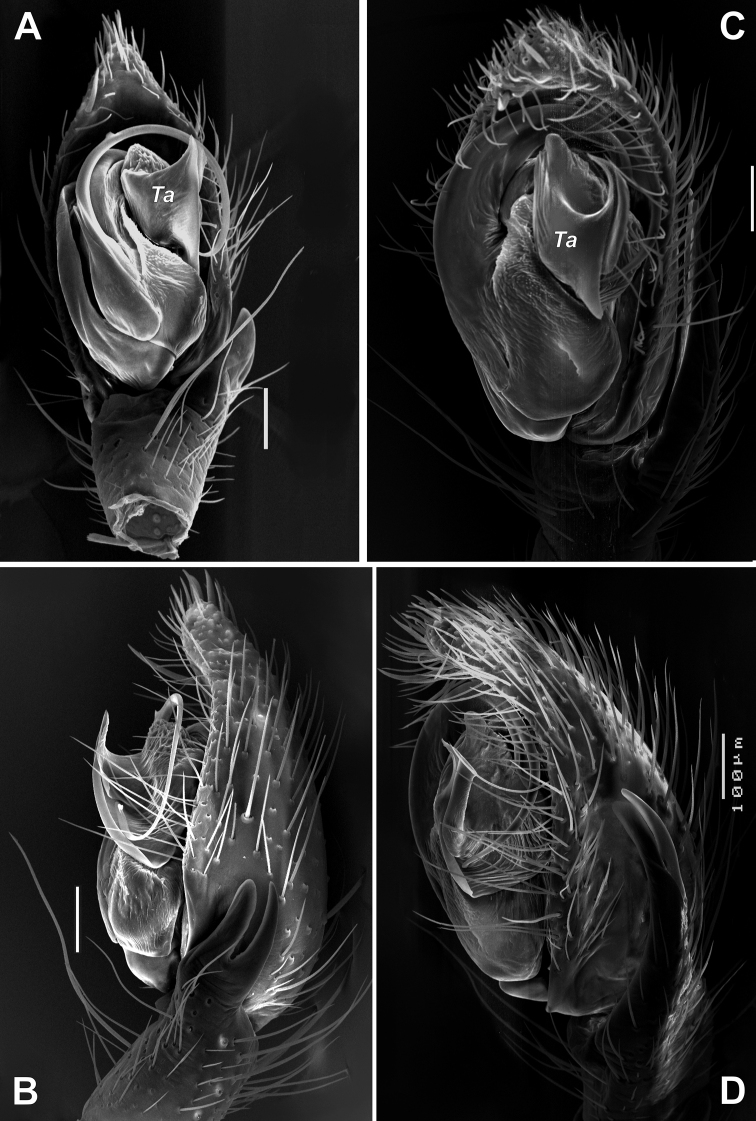
SEM images of the male palps of *Zagrotesborna* sp. nov. (**A, B**) and *Z.parla* sp. nov. (**C, D**) **A** ventral **B, D** retrolateral **C** retroventral. Abbreviation: *Ta* – tegular apophysis. Scale bars: 0.1 mm.

Figures 4A , 5A, B , 6A, B , 7A, B

##### Type material.

***Holotype*** ♂ (NHMW), Iran: Hormozgan Province: 40 km NW of Bandar Abbas, 7.4.1972 (G. Pretzmann).

##### Etymology.

The specific epithet is a Persian masculine given name meaning “young”.

##### Diagnosis.

The new species is most similar to *Z.parla* sp. nov. and can be distinguished by the RTA longer than ½ of the cymbium’s length (vs shorter) and less deeply bifurcated (cf. Fig. 6B and D), and the relatively shorter palpal tibia (4 times shorter than cymbium vs 2 times shorter), and by the shape of the bulb. The two species differ also by the shape of the sperm duct and tegular apophysis (cf. Fig. 5A and C).

##### Description.

**Male.** Habitus as in Figure 4A. Total length 4.85. Carapace 2.20 long, 1.43 wide. Eye sizes: AME: 0.11, ALE: 0.09, PME: 0.10, PLE: 0.09. Carapace, labium, chelicera, maxilla and sternum light brown, without any pattern. Legs yellowish-brown, without annulations. Abdomen cream-colored, with a tuft of dark brown long setae anteriorly and sparse lighter setae; ventrally with distinct tracheal marks. Spinnerets unicolourous. Measurements of legs: I: missing, II: 5.35 (1.45, 0.90, 1.12, 1.10, 0.78), III: 4.36 (1.20, 0.63, 0.87, 1.01, 0.65), IV: 7.06 (1.80, 1.00, 1.52, 1.81, 0.93).

Palp as in Figures 5A, B, 6A, B, 7A, B; tibia as long as wide, RTA almost twice longer than tibia, bifurcated in anterior 1/3, ventral arm rounded and dorsal one sharply pointed; cymbium 2 times longer than wide, and 4 times longer than tibia; tegular apophysis claw-like, directed laterally, with abrupt tip in lateral view; sperm duct with characteristic coil prolaterally.

**Female.** Unknown.

##### Distribution.

Known only from the type locality in Hormozgan Province, southern Iran.

#### Zagrotes parla sp. nov.

Taxon classificationAnimaliaAraneaeGnaphosidae

21203579-149A-540F-B008-F9145553B893

http://zoobank.org/0B3CDEE8-E686-4CA2-8FE8-7167C9BB0FF7

Figures 4B , 5C, D , 6C, D , 7C, D

##### Type material.

***Holotype*** ♂ (NHMW), Iran: Kerman Province: SE of Sirjan, 6.1972 (G. Pretzmann).

##### Etymology.

The specific epithet is a Persian feminine given name meaning “glowing”.

##### Diagnosis.

The new species is most similar to *Z.borna* sp. nov. and can be distinguished by the RTA shorter than ½ of the cymbium’s length (vs longer), the more deeply bifurcated RTA (cf. Fig. 6D and B), relatively longer palpal tibia (2 times shorter than cymbium vs 4 times shorter), and the shape of the bulb. The two species differ also by the shape of the sperm duct and tegular apophysis (cf. Fig. 6C and A).

##### Description.

**Male.** Habitus as in Figure 4B. Total length 4.93. Carapace 2.20 long, 1.63 wide. Eye sizes: AME: 0.10, ALE: 0.09, PME: 0.10, PLE: 0.08. Carapace, labium, chelicera, maxilla and sternum light brown, without any pattern. Legs yellowish-brown, without annulations. Abdomen cream-colored, with a tuft of dark brown long setae anteriorly and sparse lighter setae; ventrally with distinct tracheal marks. Spinnerets unicolourous. Measurements of legs: I: 6.83 (1.83, 1.17, 1.51, 1.32, 1.00), II: 5.29 (1.41, 0.93, 1.10, 1.05, 0.80), III: 4.47 (1.20, 0.70, 0.88, 1.04, 0.65), IV: 7.26 (1.84, 1.07, 1.57, 1.90, 0.88).

Palp as in Figures 5C, D, 6C, D, 7C, D; tibia 3 times shorter than cymbium; RTA as long as tibia, deeply bifurcated in middle part, ventral arm rounded on the tip and dorsal arm sharply pointed; cymbium 1.7 times longer than wide; tegular apophysis (*Ta*) with large base, about ½ of tegulum’s length, directed ventrally, sharply pointed in lateral view; sperm duct with characteristic loop originated in mesal part of tegulum.

**Female.** Unknown.

##### Distribution.

Known only from the type locality in Kerman Province, southern Iran.

###### Genus *Micaria* Westring, 1851

#### Micaria atropatene sp. nov.

Taxon classificationAnimaliaAraneaeGnaphosidae

F8C2C86A-76FA-5C80-8540-951429215094

http://zoobank.org/8E5579B5-A267-4AB2-86CA-028D610E26A5

Figures 4C , 8A, B

##### Type material.

***Holotype*** ♂ (NHMW), Iran: East Azerbaijan Province: 20 km SE of Miyaneh, 1970 (K. Bilek).

##### Etymology.

The specific epithet (a noun in apposition) refers to an ancient kingdom established in ca 323 BC by the Persian satrap Atropates, centered in present-day northern and northwestern Iran.

##### Diagnosis.

The new species is most similar to *M.rossica* (Thorell, 1875) by having a small tibial apophysis, vestigial tegular apophysis (*Ta*), a similar course of the sperm duct, and the distal tegular process (*Dp*) extending to the anterior edge of the tegulum. *Micariaatropatene* sp. nov. can be easily distinguished by the shape of the tegular process, which has an almost transverse anterior edge and a small spine-like tip (vs gradually tapering).

##### Description.

**Male.** Habitus as in Figure 4C. Total length 4.50. Carapace 2.07 long, 1.38 wide. Eye sizes: AME: 0.08, ALE: 0.08, PME: 0.05, PLE: 0.07. Carapace, labium, chelicera, maxilla and sternum reddish-brown, without any pattern. Legs yellowish-brown, without annulations. Abdomen glossy dark gray, with a coat of short setae. Spinnerets light grey, unicolourous. Measurements of legs: I: 6.01 (1.48, 0.72, 1.37, 1.21, 1.23), II: 5.28 (1.42, 0.65, 1.16, 1.00, 1.05), III: 5.01 (1.28, 0.61, 1.04, 1.11, 0.97), IV: 5.29 (1.84, 0.74, 1.44, 1.78, 1.27).

**Figure 8.**
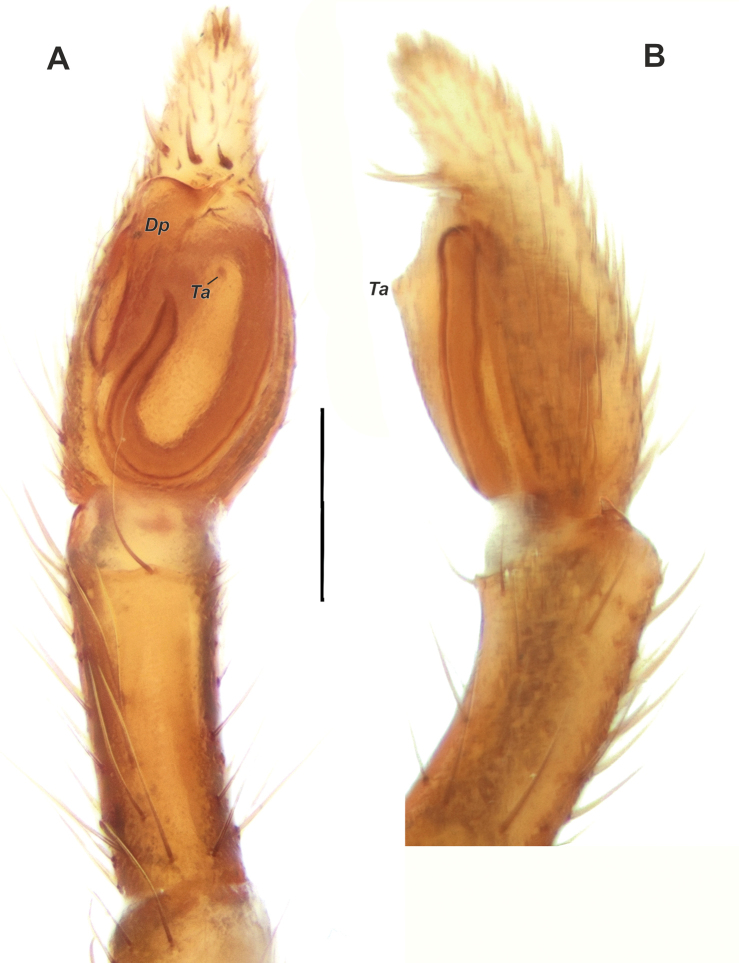
Male palp of *Micariaatropatene* sp. nov. **A** ventral **B** retrolateral. Abbreviations: *Dp* – distal tegular process, *Ta* – tegular apophysis. Scale bar: 0.2 mm.

Palp as in Figure 8A, B; tibia long, more than 3 times longer than wide, 0.83 times of cymbium’s length, with fine retrolateral apophysis; cymbium more than 2 times longer than wide; tegulum oval in ventral and lateral views, 1.8 times longer than wide; tegular apophysis (*Ta*) vestigial; distal tegular process (*Dp*) large, with abrupt anterior edge and spine-like projection mesally.

**Female.** Unknown.

##### Distribution.

Known only from the type locality in East Azerbaijan Province, northwestern Iran.

### Family Liocranidae Simon, 1897

#### Genus Sestakovaia gen. nov.

Taxon classificationAnimaliaAraneaeLiocranidae

30FBEE1C-5331-58FD-ABD8-8CF4A263D7FF

http://zoobank.org/C44399C8-5DA7-46D3-84C9-6182AA18ABB6

##### Type species.

*Sestakovaiahyrcania* sp. nov.

##### Etymology.

The new genus is named after our colleague and friend, Anna Šestáková (Western Slovakian Museum, Trnava, Slovakia); the gender is feminine.

##### Diagnosis.

**Figure 9.**
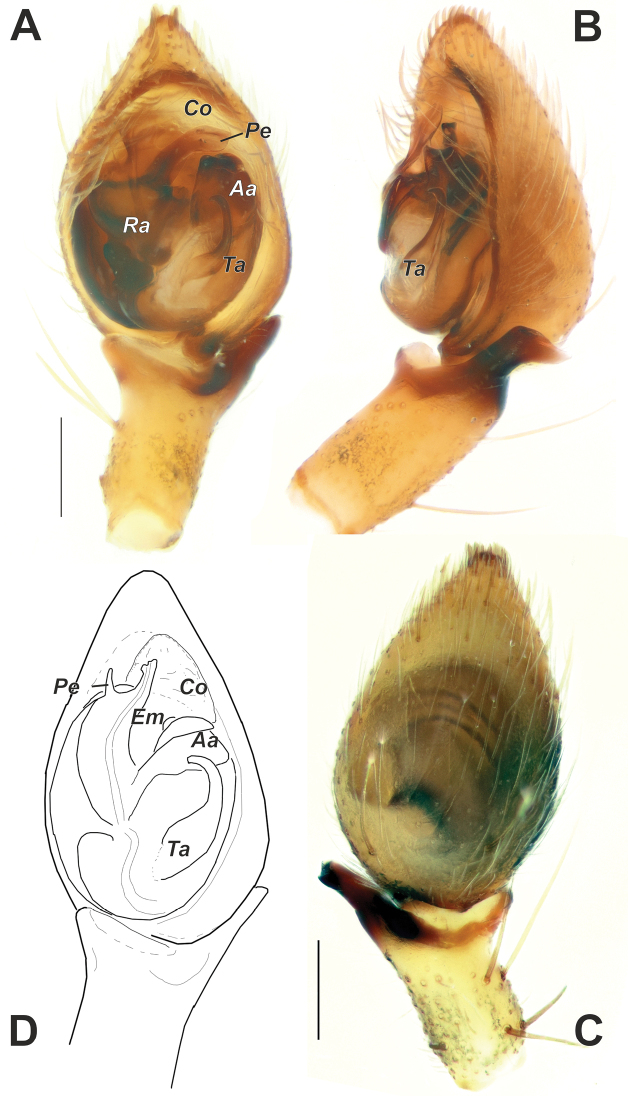
Male palps of *Sestakovaiahyrcania* sp. nov. (**A–C**) and *Sestakovaiaannulipes* comb. nov. (**D**) **A, D** ventral **B** retrolateral **C** dorsal **D** after Dimitrov and Naumova (2021). Abbreviations: *Aa* – anterior apophysis, *Co* – conductor, *Em* – embolus, *Pe* – embolic process, *Ra* – radix, *Ta* – tegular apophysis. Scale bars: 0.2 mm.

**Figure 10.**
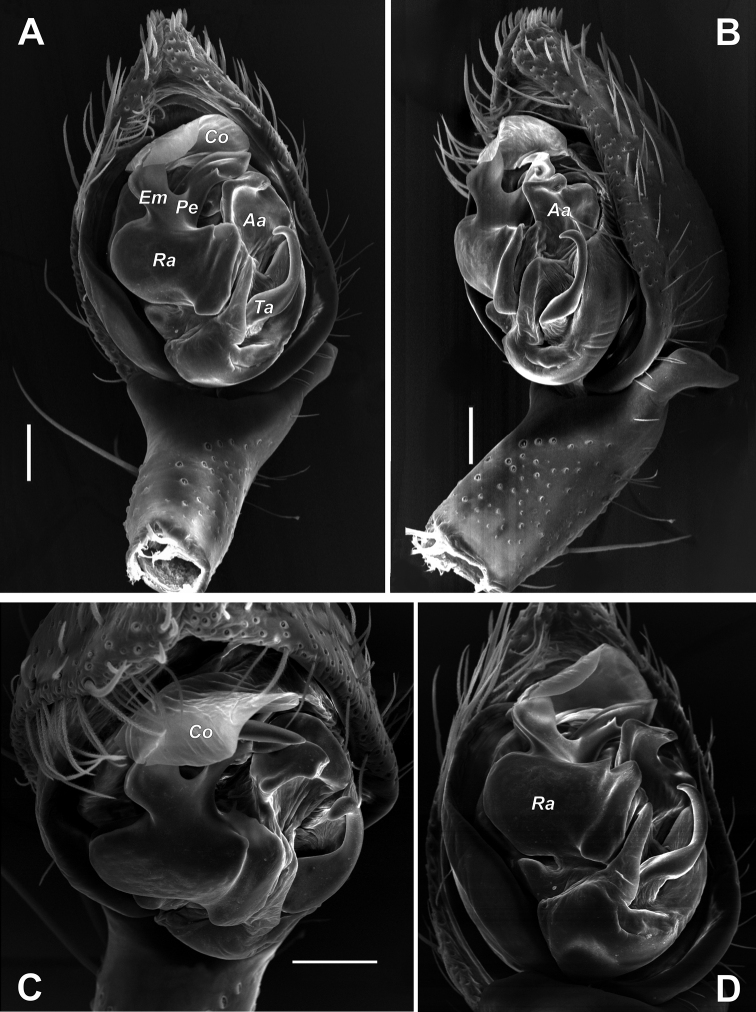
SEM images of the male palps of *Sestakovaiahyrcania* sp. nov. **A, D** ventral **B** retroventral **C** apical. Abbreviations: *Aa* – anterior apophysis, *Co* – conductor, *Em* – embolus, *Pe* – embolic process, *Ra* – radix, *Ta* – tegular apophysis. Scale bars: 0.1 mm.

**Figure 11.**
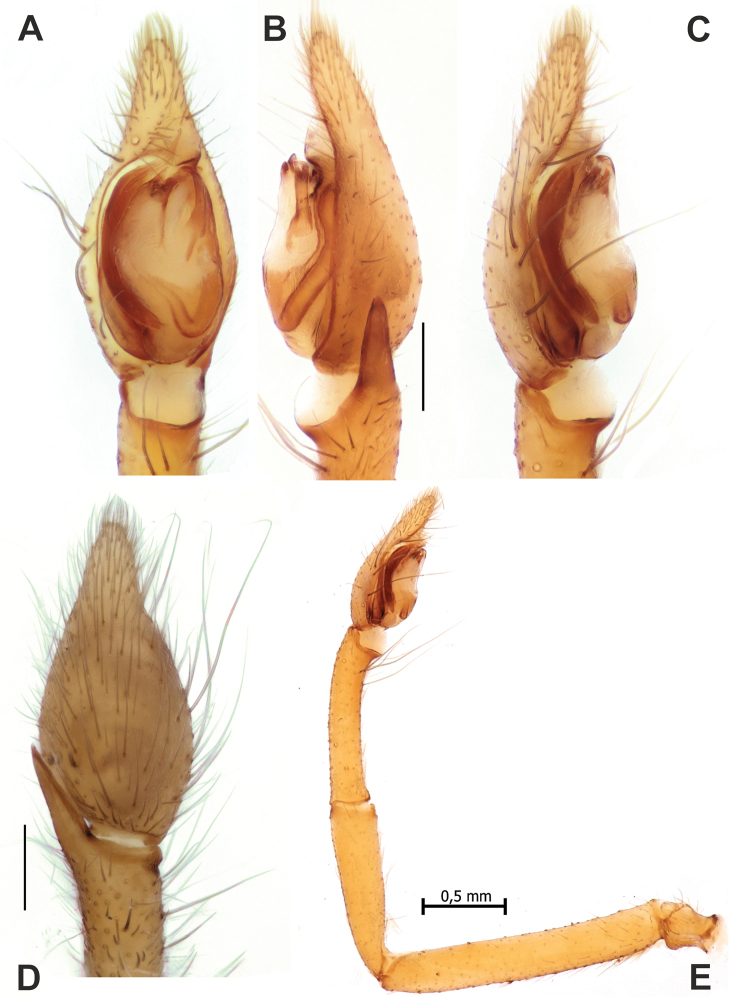
Male palp of *Mesioteluspatricki* sp. nov. **A–D** ventral, retrolateral, prolateral and dorsal, respectively **E** whole palp, prolateral. Scale bars: 0.2 mm, unless stated otherwise.

The new genus differs from other Liocraninae genera by the bent RTA (vs straight) with tip directed antero-dorsally. It differs from *Mesiotelus* Simon, 1897 by unmodified (not elongated) male chelicera, short palp (not longer than carapace), large and complex embolus (cf. Figs 9A, D, 10A with Fig. 11A) and epigyne lacking fine anterior hood (cf. Chyzer and Kulczyński 1897: pl. 9, fig. 75 with Bosmans and El-Hennawy 2018: fig. 1).

##### Description.

Medium-sized, carapace ca 2.0 long, 1.5 wide, length of chelicera ca 1/3 of carapace length. Carapace and abdomen with distinct pattern. For details, see Chyzer and Kulczyński (1897) and the description below.

##### Comments.

Homology of anterior tegular apophysis (*Aa*) is not clear, as such structure is not known in other genera of the family.

##### Composition.

*Sestakovaiahyrcania* sp. nov. and *S.annulipes* (Kulczyński, 1897), comb. nov.

##### Distribution.

Central Europe, Near East and northern Iran.

#### Sestakovaia hyrcania sp. nov.

Taxon classificationAnimaliaAraneaeLiocranidae

27BA2645-5CA6-5317-ADB6-98F2B0A0F707

http://zoobank.org/FEA79A4C-71F9-4080-8EEE-A02F4937F08E

Figures 4D , 9A–C , 10A–D

##### Type material.

***Holotype*** ♂ (NHMW), Iran: Golestan Province: 10 km SW of Shahpasand, 26.4.1972 (G. Pretzmann).

##### Etymology.

The species name (a noun in apposition) refers to the historical region in south-east of the Caspian Sea in modern-day Iran and Turkmenistan which lies between the coastal line to the north-west, the Alborz Mountains to the south, and the Kopet Dag Mountains to the east, in which the type locality of the new species is situated.

##### Diagnosis.

The new species differs from *S.annulipes* comb. nov. by having the RTA bent almost at a right angle and with a pointed tip (vs RTA bent at a lesser angle and its tip rounded), a wider embolus (*Em*) and process of the embolus (*Pe*), and the embolus located prolaterally in relation to the process (vs embolus mesal and process prolateral).

##### Description.

**Male.** Habitus as in Figure 4D. Total length 4.37. Carapace 2.06 long, 1.58 wide. Eye sizes: AME: 0.13, ALE: 0.11, PME: 0.09, PLE: 0.12. Carapace, labium, chelicera, maxilla and sternum light brown. Carapace with darker submarginal longitudinal bands. Legs yellowish-brown, with numerous darker patches. Abdomen dorsally greyish with lighter chevron markings, ventrally lighter without any markings. Spinnerets light grey, unicolourous. Measurements of legs: I: 6.54 (1.72, 0.85, 1.69, 1.32, 0.96), II: 5.73 (1.31, 0.66, 1.66, 1.30, 0.80), III: 5.82 (1.46, 0.71, 1.38, 1.63, 0.64), IV: 8.25 (2.10, 0.88, 1.96, 2.25, 1.06).

Palp as in Figures 9A–C, 10A–D; RTA about 2 times shorter than tibia, bent almost at a right angle, tip pointed; cymbium 1.6 times longer than wide; bulb ca 1.3 times longer than wide; tegulum with 2 apophyses: long tegular apophysis (*Ta*) with hooked tip, and broad anterior apophysis (*Aa*); conductor (*Co*) lamellar/membranous, large, almost as wide as radix; embolus complex: with broad radical part (*Ra*), broad embolus proper (*Em*) and mesal embolic process (*Pe*).

**Female.** Unknown.

##### Distribution.

Known only from the type locality in Golestan Province, northern Iran.

#### Sestakovaia annulipes

Taxon classificationAnimaliaAraneaeLiocranidae

(Kulczyński, 1897) comb. nov.

F3BDD7A6-8623-5203-B8FC-8E01DBBA6D28

Figure 9D

Liocranum annulipes Kulczyński in Chyzer and Kulczyński 1897: 240, pl. 9, figs 54, 56 (♂♀).
Mesiotelus annulipes : Dimitrov and Naumova 2021: 695, figs 5A, B, 6A, B (♂).

##### Comments.

This species transferred to this new genus due to the similarity of the male palp to the generotype. Although this species has a relatively large range (reported from Slovakia, Hungary, Croatia, Serbia, Bulgaria, Ukraine and Turkey; WSC 2021), the female characters have not been redescribed and the structure of the endogyne remains unillustrated.

###### Genus *Mesiotelus* Simon, 1897

#### Mesiotelus patricki sp. nov.

Taxon classificationAnimaliaAraneaeLiocranidae

E33785A3-305F-56E0-9A7F-111E39BB59CB

http://zoobank.org/3D4AD9D0-D4D9-4097-86FC-983C99FC643C

**Figure 12.**
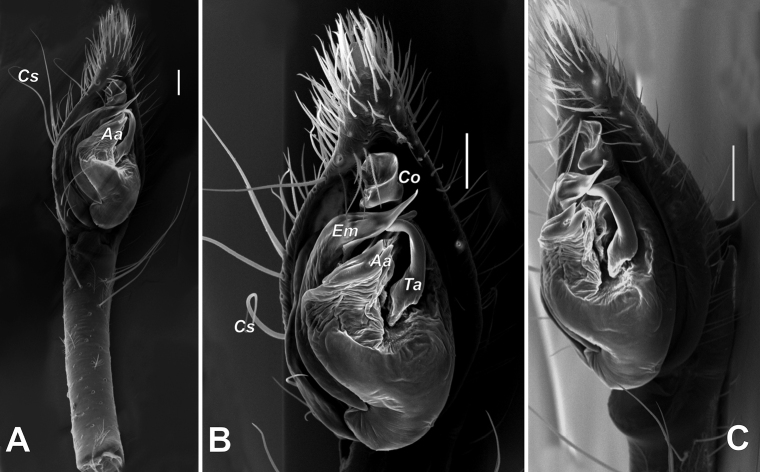
SEM images of the male palp of *Mesioteluspatricki* sp. nov. **A, B** ventral **C** retroventral. Abbreviations: *Aa* – anterior apophysis, *Co* – conductor, *Cs* – curved setae, *Em* – embolus, *Ta* – tegular apophysis. Scale bars: 0.1 mm.

Figures 4E, F , 11A–E , 12A–C

##### Type material.

***Holotype*** ♂ and ***paratypes*** 2♂ (MHNG), Iran: Golestan Province: Gol-e Loweh, 37°20'N, 55°44'E, 21.8.1975 (A. Senglet).

##### Etymology.

This new species is named after our colleague and friend L. Brian Patrick (Dakota Wesleyan University, USA), in recognition of his efforts in popularizing taxonomy via his podcast “New Species”.

##### Diagnosis.

The new species differs from the generotype, *Mesiotelustenuissimus* (L. Koch, 1866), by having a relatively longer, thinner and gradually tapering RTA which is about 3 times longer than its basal width (vs RTA with subparallel margins and less than 2 times longer than wide), a much longer median apophysis, about ½ of tegulum’s length (vs about 4 times shorter than tegulum), and a longer embolus terminating anteriorly from the tegulum (vs embolus short, spine-like and terminating beyond the anterior edge of the tegulum). The two species also differ by the course of sperm duct and the shape of the anterior tegular apophysis (*Aa*).

##### Description.

**Male (*holotype*).** Habitus as in Figure 4F. Total length 5.22. Carapace 2.00 long, 1.85 wide. Eye sizes: AME: 0.12, ALE: 0.13, PME: 0.10, PLE: 0.10. Carapace, labium, chelicera, maxilla and sternum light brown. Chelicera with 2 pro- and 3 retromarginal teeth (Fig. 4E). Legs yellowish-brown, without annulations. Abdomen dark grey, dorsally with indistinct chevron markings, ventrally lighter with distinct tracheal marks. Spinnerets light grey, unicolourous. Measurements of palp and legs: palp: 4.77 (1.74, 1.08, 1.08, 0.87), legs: I: 10.86 (2.77, 1.39, 2.95, 2.45, 1.30), II: 7.67 + missing tarsus (2.31, 1.08, 2.30, 1.98, missing), III: 7.38 (1.94, 0.91, 1.67, 1.95, 0.91), IV: 10.38 (2.63, 1.10, 2.48, 3.06, 1.11).

Palp as in Figures 11A–E, 12A–C; palp very long, almost as long as body, patella as long as tibia; tibia with 3 times longer than wide and gradually tapering RTA; cymbium twice shorter than femur, and 2 times longer than wide, tip as long as RTA, prolateral side with few very long and curled setae (*Cs*, Fig. 12A, B); bulb oval, 1.67 times longer than wide; sperm duct almost reaching posterior margin of tegulum; median apophysis long, almost as long as ½ of tegulum’s length; anterior tegular apophysis (*Aa*) bifid; embolus (*Em*) originates at about ca 6:30 o’clock position, free part long, bent in terminal part and directed anteriorly.

**Female.** Unknown.

##### Distribution.

Known only from the type locality in Golestan Province, northern Iran.

### Family Palpimanidae Thorell, 1870

#### Genus Palpimanus

Taxon classificationAnimaliaAraneaePalpimanidae

Dufour, 1820

86C159AF-F28F-55FE-8166-CD0EE78CE4E2

##### Comments.

*Palpimanus* is the most species-rich genus in Palpimaninae, with 35 currently recognized species (WSC 2021). Although the Mediterranean species have been the subject of two revisions by Kulczyński (1909) and Platnick (1981), the genus remains poorly studied: male palps were illustrated very schematically and endogynes were not illustrated at all. Additionally, there is no proper terminology for the sclerites in the male palp; Platnick (1981) used the neuter terms ‘prong’ and ‘flange’. Within Palpimaninae, the endogynes are very weakly sclerotized and difficult to observe, hence the lack of proper illustrations. The set of illustrations in Platnick (1981: figs 10–18) is very schematic and in some cases appears to be misinterpreted. Both new species found in Iran have male palps rather different from that of the type species (*P.gibbulus* Dufour, 1820) and most likely belong to a different, currently undescribed genus.

#### Palpimanus carmania sp. nov.

Taxon classificationAnimaliaAraneaePalpimanidae

4DAED8F0-3268-530D-96D0-CC8459C1DF40

http://zoobank.org/868D7059-4D36-47BB-9C6B-45B3065CB57B

**Figure 13.**
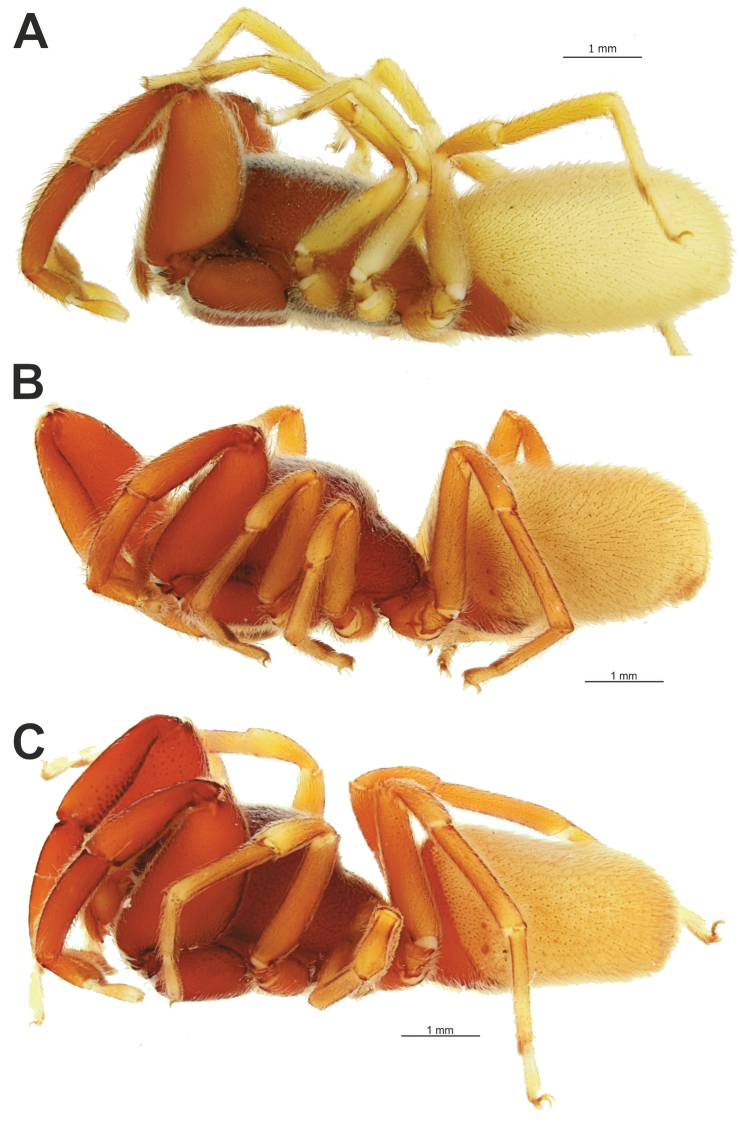
Lateral habitus of *Palpimanuscarmania* sp. nov. (**A**) and *P.persicus* sp. nov. (**B, C**) **A, C** males **B** female.

**Figure 14.**
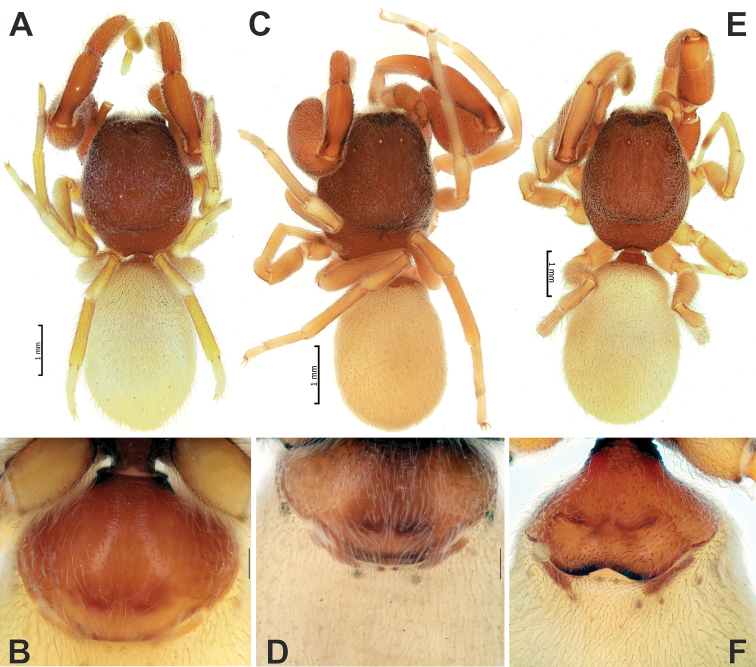
Dorsal habitus (**A, C, E**) and ventral anterior portion of abdomen (**B, D, F**) of *Palpimanuscarmania* sp. nov. (**A, B**) and *P.persicus* sp. nov. (**C–F**) **A–D** males **E, F** female. Scale bars: 0.2 mm, unless stated otherwise.

**Figure 15.**
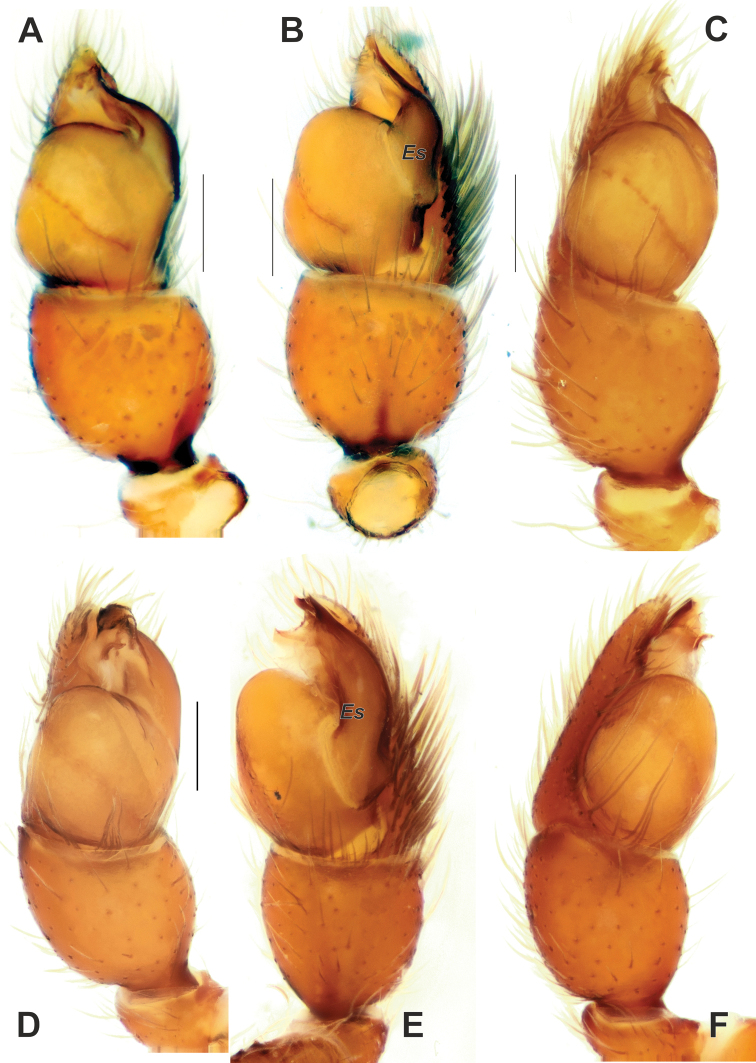
Male palps of *Palpimanuscarmania* sp. nov. (**A–C**) and *P.persicus* sp. nov. (**D–F**) **A, D** ventral **B, E** retrolateral **C, F** proventral and prolateral. Abbreviation: *Es* – embolic stalk. Scale bars: 0.2 mm.

**Figure 16.**
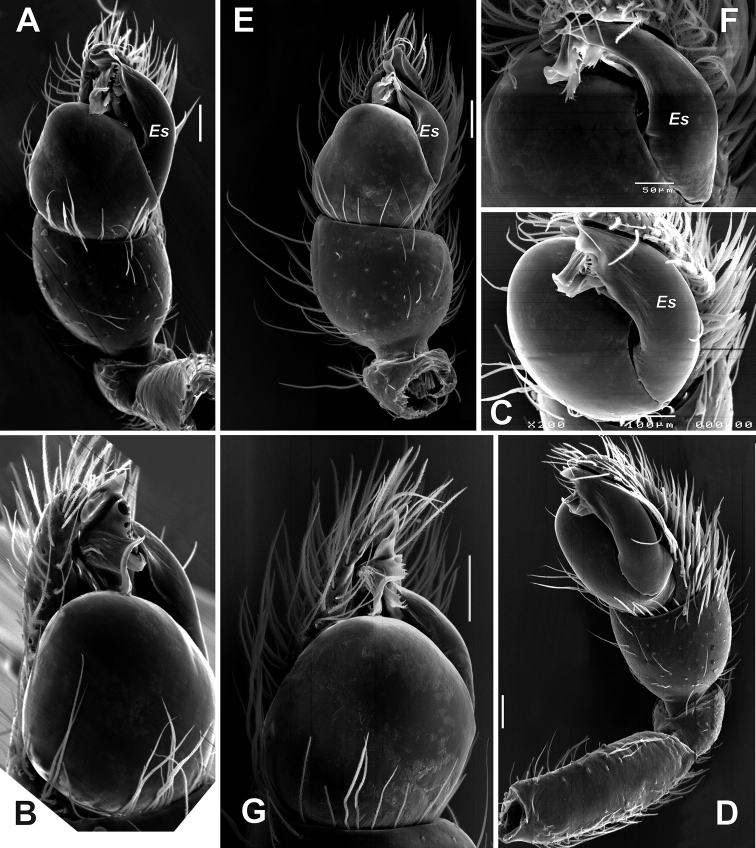
SEM images of the male palps of *Palpimanuspersicus* sp. nov. (**A–D**) and *P.carmania* sp. nov. (**E–G**) **A, E** ventral **B, G** proventral **C, F** apical **D** proapical. Abbreviation: *Es* – embolic stalk. Scale bars: 0.1 mm, unless stated otherwise.

Figures 13A , 14A, B , 15A‒C , 16E‒G

##### Type material.

***Holotype*** ♂ (NHMW), Iran: Kerman Province: 41 km SE of Sirjan, 18.4.1972 (G. Pretzmann).

##### Etymology.

The specific epithet is a noun in apposition, referring to a historical region that approximately corresponds to the modern Iranian province of Kerman, where the type locality of the new species is situated.

##### Diagnosis.

The new species differs from the related *P.persicus* sp. nov. by the more distal position of the base of the “embolic stalk” (*Es*) and wider stalk with sharply pointed tip (vs more proximal, thinner and without sharply pointed tip; cf. Fig. 16E and A) and also by having wider than long palpal tibia (vs as wide as long). It is most similar to P.cf.sogdianus from Azerbaijan illustrated by Marusik and Guseinov (2003). Both have a sharply pointed embolic stalk (*Es*), but in the new species this originates from the distal half of the bulb (vs proximal half).

##### Description.

**Male.** Habitus as in Figures 13A, 14A. Total length 5.50. Carapace 2.65 long, 1.92 wide. Eye sizes: AME: 0.17, ALE: 0.08, PME: 0.07, PLE: 0.10. Carapace, labium, chelicera, maxilla and sternum dark reddish, coated with white setae. Leg I dark orange, legs II‒IV yellowish-brown, without annulations. Metatarsus I and metatarsi and tarsi II‒IV with a distinct ventral hair tuft. Abdomen cream-colored, with scattered long dark setae and a ventral scutum, two long diagonal and two dot-like scutula (Fig. 14B). Spinnerets unicolourous. Measurements of legs: I: 6.05 (1.90, 1.58, 1.34, 0.58, 0.65), II: 4.59 (1.26, 0.90, 1.12, 0.74, 0.57), III: 4.05 (1.16, 0.80, 0.87, 0.76, 0.46), IV: 5.18 (1.35, 0.86, 1.28, 1.08, 0.61).

Palp as in Figures 15A‒C, 16E‒G; tibia swollen, ca 1.1 times wider than long, wider than bulb; cymbium 1.5 times longer than tibia; embolic stalk (*Es*) originates in anterior half, stalk tapering, tip sharply pointed.

**Female.** Unknown.

##### Distribution.

Known only from the type locality in Kerman Province, southern Iran.

#### Palpimanus persicus sp. nov.

Taxon classificationAnimaliaAraneaePalpimanidae

B32FC728-E19A-567E-A0FD-93C44C9CBA28

http://zoobank.org/966664CC-AAFC-42AC-B3F8-D0BD28E26BA2

**Figure 17.**
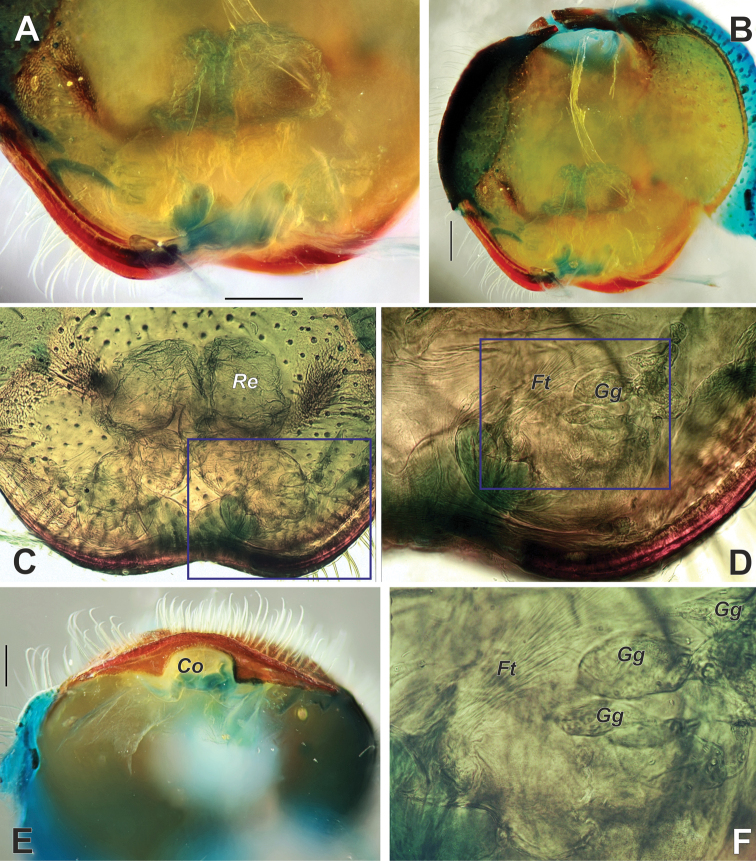
Endogyne of *Palpimanuspersicus* sp. nov. **A–D, F** dorsal **E** posterior. Boxes in **C** and **D** refer to detailed views presented in **D** and **F**, respectively. Abbreviations: *Co* – copulatory opening, *Ft* – fine threads, *Gg* – grape-shaped glands, *Re* – receptacle. Scale bars: 0.2 mm.

Figures 13B, C , 14C‒F , 15D‒F , 16A‒D , 17A‒F

##### Type material.

***Holotype*** ♂ (NHMW), Iran: Hormozgan Province: 40 km NW of Bandar Abbas, 7.4.1972 (G. Pretzmann). ***Paratype***: 1♂ (NHMW), Hormozgan Province: 26 km S of Minab, 7.1974 (G. Pretzmann).

##### Other material examined.

Iran: Hormozgan Province: 1♀ (NHMW), 28 km N of Bandar Abbas, 28.3.1972 (G. Pretzmann).

##### Etymology.

The specific epithet is an adjective of “Persian” or “of Persia”, referring to the historical region of the Middle East, located in the east of Mesopotamia (nowadays Iran).

##### Diagnosis.

The male of this species differs from the closely related *Palpimanuscarmania* sp. nov. by having the embolic stalk originating in the proximal half of the bulb (vs distal) and the stalk wider, not tapering, and its tip not pointed (cf. Fig. 16A and E). Endogyne of the new species is similar to that of *P.sogdianus* Charitonov, 1946 and specimens identified as P.cf.sogdianus from Azerbaijan (see Marusik and Guseinov 2003: figs 35–36), but it differs by having the anterior and posterior halves of the receptacles subequal in length (vs anterior halves longer). In addition, females of the new species differ from specimen from Azerbaijan by having the copulatory opening (*Co*) more arched.

##### Description.

**Male (*holotype*).** Habitus as in Figures 13C, 14C. Total length 5.20. Carapace 2.60 long, 1.95 wide. Eye sizes: AME: 0.21, ALE: 0.11, PME: 0.07, PLE: 0.13. Carapace, labium, chelicera, maxilla and sternum dark reddish, coated with white setae. Leg I dark orange, legs II‒IV yellowish-brown, without annulations. Metatarsus I and metatarsi and tarsi II‒IV with a distinct ventral hair tuft. Abdomen cream-colored, with scattered long dark setae and a ventral scutum, two long diagonal and two dot-like scutula (Fig. 14D). Spinnerets unicolourous. Measurements of legs: I: 5.57 (1.77, 1.46, 1.27, 0.57, 0.50), II: 4.74 (1.44, 0.92, 1.10, 0.72, 0.56), III: 4.23 (1.29, 0.76, 1.03, 0.70, 0.45), IV: 5.01 (1.53, 0.93, 1.20, 0.91, 0.44).

Palp as in Figures 15D‒F, 16A‒D; tibia swollen, as wide as long; cymbium 1.6 times longer than wide; embolic stalk (*Es*) originates in proximal half of bulb, wide, not tapering, tip not pointed.

**Female.** Habitus as in Figures 13B, 14E. Total length 6.55. Carapace 3.00 long, 2.27 wide. Eye sizes: AME: 0.17, ALE: 0.11, PME: 0.07, PLE: 0.11. Coloration and somatic features as in male. Postgaster with 3 pairs of scutula (Fig. 14F). Measurements of legs: I: 6.78 (2.17, 1.80, 1.62, 0.61, 0.58), II: 5.71 (1.76, 1.16, 1.32, 0.83, 0.64), III: 5.06 (1.50, 0.93, 1.17, 0.86, 0.60), IV: 6.77 (2.04, 1.19, 1.66, 1.32, 0.56).

Endogyne as in Figure 17A‒F; copulatory opening (*Co*) arched in ventral view and squared in posterior view; endogyne weakly sclerotized, receptacles about 3.5 times longer than wide, each with cylindrical posterior part and subglobular anterior part, posterior parts converging, receptacles lacking distinct accessorial or pore glands; fine threads (*Ft*) originate near copulatory opening; grape-shaped glands (*Gg*) poorly distinct, about 5 on each side.

##### Comment.

As the single female specimen treated here was collected separately from the males and in a different locality (although all from the same province and from sites relatively close to one another), it was not considered within the type material; the conspecificity of these specimens shall be confirmed when both sexes are collected together.

##### Distribution.

Known only from the listed localities in Hormozgan Province, southern Iran.

### Family Philodromidae Thorell, 1870

#### Genus Rhysodromus

Taxon classificationAnimaliaAraneaePhilodromidae

Schick, 1965

BC69C39C-DE3E-5D3A-BFA2-C007FBC58A0D

##### Comments.

Considered a relatively large genus, *Rhysodromus* includes 26 valid species, the majority of which are distributed in the Palaearctic. The genus is relatively well studied due to several revisions, especially that by Szita and Logunov (2008).

#### Rhysodromus genoensis sp. nov.

Taxon classificationAnimaliaAraneaePhilodromidae

066AD2E3-83F4-5315-8267-BFB7671EF213

http://zoobank.org/D83478E1-738C-4556-A2A5-CAAB97E559C0

**Figure 18.**
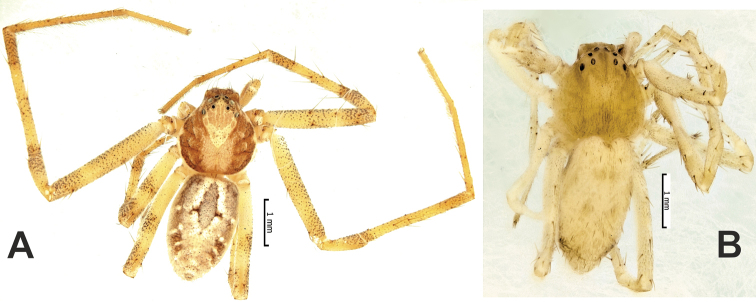
Male dorsal habitus of *Rhysodromusgenoensis* sp. nov. (**A**) and *Rhysodromusmedes* sp. nov. (**B**).

**Figure 19.**
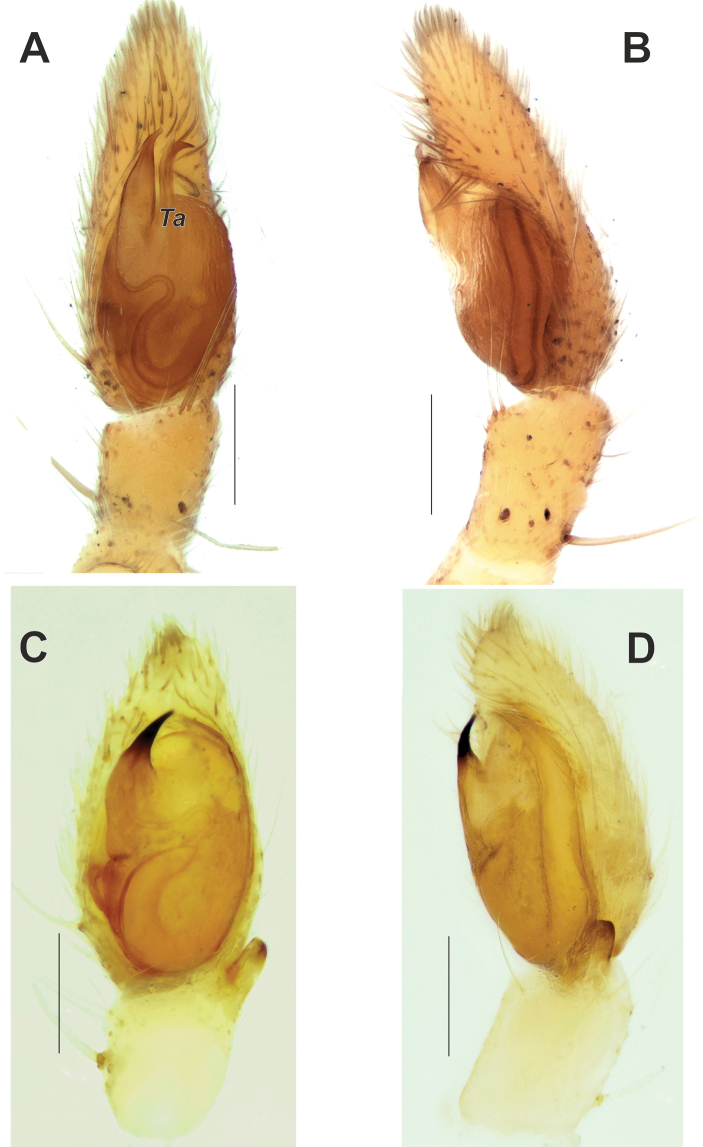
Male palps of *Rhysodromusgenoensis* sp. nov. (**A, B**) and *Rhysodromusmedes* sp. nov. (**C, D**) **A, C** ventral **B, D** retrolateral. Abbreviation: *Ta* – tegular apophysis. Scale bars: 0.2 mm.

**Figure 20.**
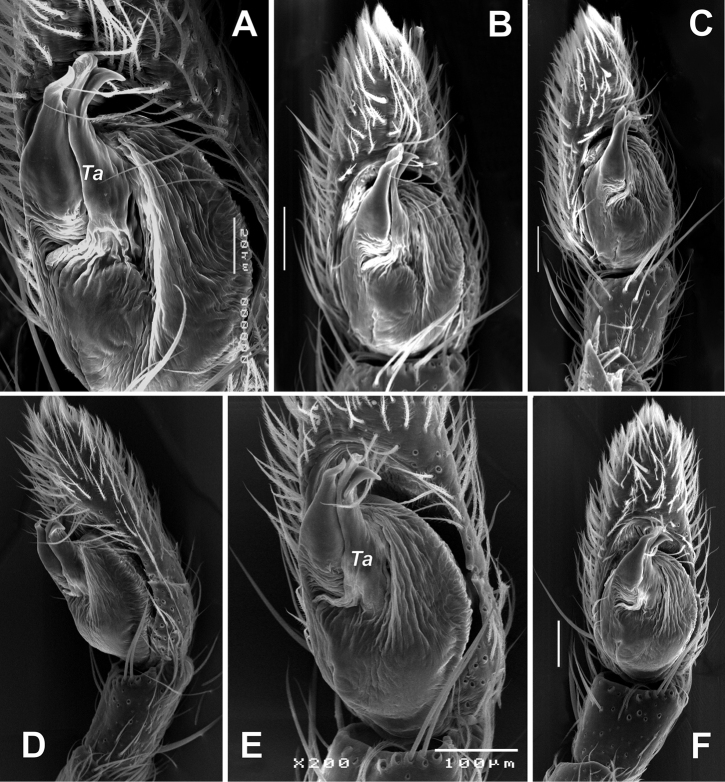
SEM images of the male palp of *Rhysodromusgenoensis* sp. nov. **A, E** retroventral **B, F** ventral **C** proventral **D** retrolateral. Abbreviation: *Ta* – tegular apophysis. Scale bars: 0.1 mm, unless stated otherwise.

Figures 18A , 19A, B , 20A‒F

##### Type material.

***Holotype*** ♂ (NHMW), Iran: Hormozgan Province: Geno, 38 km NW of Bandar Abbas, 3.4.1972 (G. Pretzmann).

##### Etymology.

The specific epithet refers to the type locality of the new species in Geno Biosphere Reserve.

##### Diagnosis.

This species differs from all congeners by the lack of the RTA (vs present in all species) and bifurcated tip of tegular apophysis (vs not bifurcated).

##### Description.

**Male.** Habitus as in Figure 18A. Total length 3.80. Carapace 1.65 long, 1.60 wide. Eye sizes: AME: 0.11, ALE: 0.10, PME: 0.06, PLE: 0.10. Carapace, labium, chelicera and maxilla brown, without any pattern; pars cephalica and median part of pars thoracica lighter in color; sternum pale, with a grayish marginal band. Legs yellowish-brown, with numerous dark spots and small patches. Abdomen grayish, dorsally with distinct cardiac mark and darker margins and light spots and patches; ventrally with slightly darker median band. Spinnerets brown, unicolourous. Measurements of legs: I: 7.86 (2.27, 0.89, 1.95, 1.74, 1.01), II: 9.15 (2.66, 0.88, 2.31, 2.05, 1.25), III: 5.55 (1.72, 0.65, 1.29, 1.16, 0.73), IV: 7.06 (2.26, 0.74, 1.69, 1.61, 0.76).

Palp as in Figures 19A, B, 20A‒F; tibia as long as bulb’s width, lacking distinct apophysis; cymbium with long tip (partially extending tegulum), about 0.9 of tegulum’s length; tegulum oval, about 1.5 times longer than wide; sperm duct with small transverse loop in mesal part of tegulum; tegular apophysis long, located anteriorly along longitudinal axis, tip bifid; embolus long, >0.5 of tegulum’s length, straight, with tip gently bent retrolaterally.

**Female.** Unknown.

##### Comments.

The new species has a pattern typical for the genus and is particularly similar to *R.alascensis* (Keyserling, 1884) and *R.histrio* (Latreille, 1819).

There are two *Rhysodromus* species in the region (both from Caucasus) known from females only, *R.rikhteri* (Logunov & Huseynov, 2008) and *R.naxcivanicus* (Logunov & Huseynov, 2008), but it is very unlikely that either of them would be conspecific with *R.genoensis* sp. nov., as their type localities are located more than 1000 km distant from that of the new species.

##### Distribution.

Known only from the type locality in Hormozgan Province, southern Iran.

#### Rhysodromus medes sp. nov.

Taxon classificationAnimaliaAraneaePhilodromidae

EC976C49-FB57-5ED1-93B4-A3F9AD30383D

http://zoobank.org/6BABB45F-31F2-4526-9CC1-9C4CFF99F9D2

**Figure 21.**
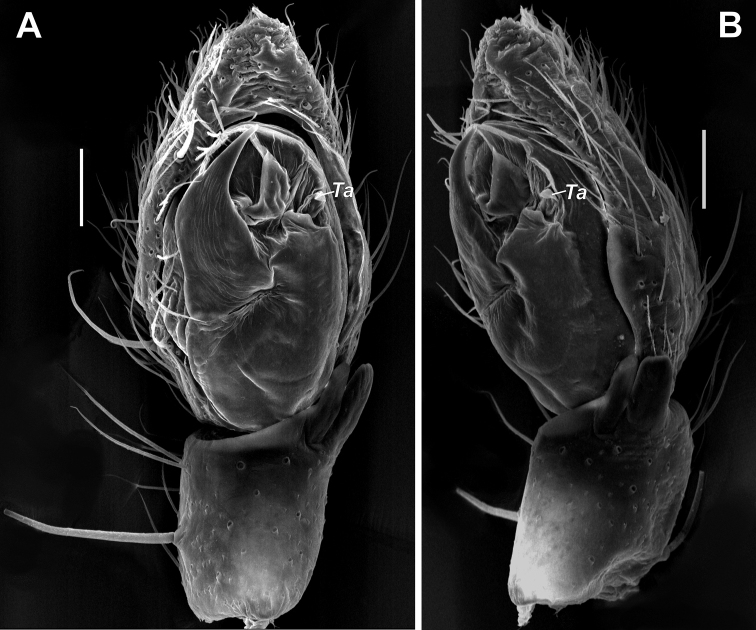
SEM images of the male palp of *Rhysodromusmedes* sp. nov. **A** ventral **B** retroventral. Abbreviation: *Ta* – tegular apophysis. Scale bars: 0.1 mm.

Figures 18B , 19C, D , 21A, B

##### Type material.

***Holotype*** ♂ (NHMW), Iran: Hormozgan Province: 26 km S of Minab, 7.1974 (G. Pretzmann).

##### Etymology.

The specific epithet is a noun in apposition, referring to an ancient Iranian people who spoke the Median language and inhabited an area known as Media between western and northern Iran.

##### Diagnosis.

The new species differs from all congeners (as well as all other members of *Philodromus* sensu lato) by having 2 parallel, unspaced tibial apophyses of the same length (vs apophyses either absent, or 1 or 2 spaced and not of equal length) and sperm duct thick at proximal-prolateral part, as thick as retrolaterally (vs prolateral part gradually tapering and thinner than retrolateral part).

##### Description.

**Male.** Habitus as in Figure 18B. Total length 4.07. Carapace 1.87 long, 1.60 wide. Eye sizes: AME: 0.11, ALE: 0.07, PME: 0.10, PLE: 0.11. Carapace, labium, chelicera, maxilla and sternum yellowish-brown. Legs pale, without annulations. Abdomen pale, with scattered thick dark setae. Spinnerets pale, unicolourous. Measurements of legs: I: 8.97 (2.52, 0.96, 2.41, 2.06, 1.02), II: 11.58 (3.20, 1.19, 3.10, 2.78, 1.31), III: 4.97 (1.52, 0.52, 1.07, 1.18, 0.68), IV: 8.54 (2.65, 0.75, 1.95, 2.23, 0.96).

Palp as in Figures 19C, D, 21A, B; tibia ca 1.3 times longer than wide, with two apophyses, not spaced and equal in length, about ½ of tibia’s length, ventral one membranous and transparent, retrolateral one well sclerotized; cymbium 1.7 times longer than wide, with tip equal in length to tibial apophyses; tegulum oval, ca 1.8 times longer than wide; sperm duct thick, only 2.5 times thinner than tibia’s diameter, proximal-prolateral part as thick as retrolateral part, sharply tapering in distal half of tegulum; tegular apophysis (*Ta*) small, claw-like, located antero-retrolaterally; embolus large, about 0.63 of tegulum’s length, base as wide as ½ of tegulum, gradually tapering.

**Female.** Unknown.

##### Comments.

This species is tentatively placed in *Rhysodromus*, as it has a different pattern and a very thick sperm duct which is unknown in other members of the genus. The generic placement is due to the presence of tegular apophysis and two closely placed tibial apophyses, similar to *R.mysticus* (Dondale & Redner, 1975).

##### Distribution.

Known only from the type locality in Hormozgan Province, southern Iran.

## Supplementary Material

XML Treatment for Brigittea

XML Treatment for Brigittea avicenna

XML Treatment for Zagrotes

XML Treatment for Zagrotes borna

XML Treatment for Zagrotes parla

XML Treatment for Micaria atropatene

XML Treatment for Sestakovaia

XML Treatment for Sestakovaia hyrcania

XML Treatment for Sestakovaia annulipes

XML Treatment for Mesiotelus patricki

XML Treatment for Palpimanus

XML Treatment for Palpimanus carmania

XML Treatment for Palpimanus persicus

XML Treatment for Rhysodromus

XML Treatment for Rhysodromus genoensis

XML Treatment for Rhysodromus medes
